# Trigeminal Autonomic Cephalalgias: Beyond the Conventional Treatments

**DOI:** 10.1007/s11916-014-0438-z

**Published:** 2014-06-29

**Authors:** Sarah Miller, Manjit Matharu

**Affiliations:** 1 Headache Group, Institute of Neurology, Queen Square, London, WC1N 3BG UK; 2 The National Hospital for Neurology and Neurosurgery, Queen Square, London, WC1N 3BG UK

**Keywords:** Trigeminal autonomic cephalalgias, Neurostimulation, Nerve blocks, Sphenopalatine ganglion stimulation, Occipital nerve stimulation, Deep brain stimulation

## Abstract

The trigeminal autonomic cephalalgias include cluster headache, paroxysmal hemicrania, short-lasting unilateral neuralgiform headache attacks, and hemicrania continua. While the majority responds to conventional pharmacological treatments, a small but significant proportion of patients are intractable to these treatments. In these cases, alternative choices for these patients include oral and injectable drugs, lesional or resectional surgery, and neurostimulation. The evidence base for conventional treatments is limited, and the evidence for those used beyond convention is more so. At present, the most evidence exists for nerve blocks, deep brain stimulation, occipital nerve stimulation, sphenopalatine ganglion stimulation in chronic cluster headache, and microvascular decompression of the trigeminal nerve in short-lasting unilateral neuralgiform headache attacks.

## Introduction

Trigeminal autonomic cephalalgias (TACs) are primary headache disorders characterized by unilateral head pain occurring in association with ipsilateral cranial autonomic symptoms. TACs include cluster headache (CH), paroxysmal hemicrania (PH), and short-lasting unilateral neuralgiform headache attacks [including both short-lasting unilateral neuralgiform headache attacks with conjunctival injection and tearing (SUNCT), and short-lasting unilateral headache attacks with cranial autonomic symptoms (SUNA)], and hemicrania continua (HC) [1]. The clinical features of these attacks and their response to medications are often distinctly characteristic (Table 1).

**Table 1:** Clinical features of the trigeminal autonomic cephalalgias and first-line treatments

	Cluster headache	Paroxysmal hemicrania	Hemicrania continua	Short-lasting unilateral neuralgiform headache attacks (SUNCT/SUNA)
ICHD-3 beta Code	3.1	3.2	3.4	3.3 SUNCT 3.3.1 SUNA 3.3.2
Sex (F:M)	1:5–7	1:1	2:1	1:2
Pain Type Severity	Stabbing, boring Severe	Throbbing, stabbing, boring Moderate–severe	Throbbing, ache, sharp, pressure Moderate background with severe exacerbations	Sharp, shooting, burning Severe
Duration and frequency	15–180 mins, 1–8/day	2–30 mins, 5–40/day	Continuous	1–600 s, 1–200/day
Autonomic features	Yes	Yes	Yes with exacerbations	Yes^a^
Indometacin effect	No	Complete resolution	Complete resolution	No
First choice abortive agent	Injectable or nasal sumatriptan Oxygen	Nil	Nil	Nil
First choice preventative agents	Verapamil Lithium Topiramate Methysergide^b^	Indometacin	Indometacin	Lamotrigine

*SUNCT* short-lasting unilateral neuralgiform headache attacks with conjunctival injection and tearing, *SUNA* short-lasting unilateral neuralgiform headache attacks with autonomic symptoms, *ICHD-3 beta* International Classification of Headache Disorders 3rd edition Beta version, *F* female, *M* male

^a^SUNCT: both conjunctival injection and tearing; SUNA: only one or neither of conjunctival injection and tearing

^b^Methysergide is either not available or is due to be withdrawn in several countries

The majority of patients will respond to standard or first-line treatments, as outlined in international guidelines (Table 1) [2]. A small, but highly disabled, group of patients will fail to respond to these treatments, and this has led to the search for alternative treatments.

Standard treatment recommendations for TACs are based on moderate-to-low quality evidence, with little in the way of randomized trials. These will not be covered in this article but were recently reviewed by Pareja and Alvarez [3••]. Nonconventional treatments are used on the basis of clinical experience and open-label series. The nonconventional treatments currently in clinical use are reviewed here.

## CH

CH consist of attacks of severe, strictly unilateral pain occurring in the orbital, supraorbital and temporal regions, lasting 15–180 minutes, and that occur from once every other day to eight times daily [1]. The pain is associated with autonomic features and agitation (Table 1).

### Acute Treatment

The conventional acute treatments for CH attacks are shown in Table 1. When these fail, other options are scarce. Intranasal lidocaine has limited evidence [4], and subcutaneous octreotide, a somatostain analogue, was found to be superior to placebo in a single study [5]. Owing to practicalities of administration, both have limited clinical usefulness.

### Preventative Treatment

International guidelines give verapamil, lithium, methysergide, topiramate, valproate, melatonin, and baclofen as first-line treatments [2]. In their review, Pereja and Alvarez describe verapamil, lithium, and topiramate as being the standard treatments most commonly in use [3••]. Below, we shall provide a summary of treatments aside from verapamil, lithium, methysergide, and topiramate (Table 2).

**Table 2:** Summary of evidence for oral and injectable alternative treatments for cluster headache

	*n*	Outcome	Dosing details
Gabapentin			
Leandri et al. [6], Schuh-Hofer et al. [7]	20 (12 CCH)	Positive 18/20	800–3600 mg daily
Melatonin			
Leone et al. [10]	10 (treatment group; 2 CCH), 10 (placebo group)	Treatment group: positive 5/10 treated Placebo group: positive 0/10 Significant difference to placebo (*p* < 0.03)	10 mg daily
Pringsheim et al. [11]	9 (6 CCH), crossover trial	No significant difference to placebo (*p* > 0.05)	2 mg daily for 1 month, placebo for one month
Baclofen			
Hering-Hanit et al. [12]	16 (ECH)	Positive 13/16	15–30 mg daily
Valproic acid			
Hering et al. [13]	15 (2 CCH)	Positive 11/15	600–2000 mg daily
El Amrani et al. [14]	50 (treatment group; 11 CCH), 46 (placebo group; 6 CCH)	Treatment group: positive 25/50 Placebo group: positive 29/50 No significant difference to placebo (*p* = 0.23)	1–2 g daily
Candesartan			
Tronvik et al. [15]	24 (treatment group), 16 (placebo group)	No significant difference to placebo for change in attack frequency (*p* = 0.38). Post hoc analysis significant difference to placebo (*p* < 0.01, RR 1.3)	32 mg daily
Levitracetam			
Palermo et al. [16]	2	2	1–2 g (unpublished data on further 3 CCH responding)
Botulinum toxin			
Sostak et al. [17], Robbins [18], Freund and Schwartz [19], Smuts and Barnard [20]	24 (15 CCH)	Positive 11/24	24–50 units
Nerve blocks			
GON			
Leurox et al. [22•]	21 (treatment group; 7 CCH), 22 (placebo group; 8 CCH)	Significant difference between groups in mean no attacks a day until day 15 (*p* < 0.01) No significant difference between groups reaching 50 % reduction of attacks at 15 days (*p* = 0.06)	Treatment: 3.75 mg cortivazol, 3 injections given 48–72 h apart Placebo: saline
Ambrosini et al. [23•]	13 (treatment group; 4 CCH), 10 (placebo group; 3 CCH)	Treatment group: positive 11/13 (4 CCH) Placebo group: positive 0/10 Significant difference between groups (*p* < 0.01)	Treatment: betamethasone and xylocaine Placebo: saline and xylocaine
Lambru et al. [83]	83 (83 CCH)	Positive 47/83 (first injection), 31/37 (second injection), 27/28 (third injection)	80 mg methylprednisolone and lidocaine
SPG			
Pipolo et al. [25]	15 (15 CCH)	Positive 8/15	Triamcinolone acetonide, bupivacane, mepivacane, and 1/100,000 adrenaline injected with endoscopic guidance

*CCH* chronic cluster headache, *ECH* episodic cluster headache, *RR* relative risk, *GON* greater occipital nerve, *SPG* sphenopalatine ganglion

#### Oral Treatments

Gabapentin is widely used in the treatment of pain and has therefore been trialled in CH with some highly positive data in a limited series of patients. In the series of Leandri et al. [6], 12 patients undertook an open trial of gabapentin. All reported freedom from pain after 8 days of treatment. Schuh-Hofer et al. [7] used gapapentin as an adjunctive treatment in eight patients with chronic CH (CCH) who had failed first-line therapies. Six of eight patients reported a more than 50 % reduction in attack frequency, with two claiming pain freedom at the 4-months follow-up. Doses used by this group ranged from 800 to 3600 mg. Long-term data on the use of gabapentin do not exist, but Schuh-Hofer et al. [7] saw that, over a year, follow-up patients did seem to develop tolerance. Although the authors state that gabapentin is a well-tolerated drug the dosage of which can be rapidly increased over a short period of time, in clinical practice their highly positive rapid-onset results have not been observed. It is relevant to note that although gabapentin was previously considered beneficial for migraine, both a placebo-controlled study by Silberstein et al. [8] and recent Cochrane review have found no evidence of efficacy [9].

Melatonin has been tried in CH, but there are contradictory findings. Leone et al. [10] found a significant reduction in headache frequency in their 2-week randomized placebo controlled trial, with 5/10 patients with episodic CH (ECH) treated reporting cessation of their attacks after 5 days of treatment (10 mg daily) but no effect on patients with CCH. Pringsheim et al. [11] conducted a placebo-controlled crossover study using 2 mg daily for 1 month in nine patients. They reported no significant difference between melatonin and placebo. Although conflicting evidence exists, melatonin makes for an attractive therapy given its lack of side effects and drug interactions. An argument can be made that the negative trial group used a very low dose and, in practice, our unit recommends a treatment dose of 12–16 mg daily. Further trials with higher doses need to be carried out before clear recommendations can be made.

Baclofen has gamma-aminobutyric acid-ergic actions and is used in a variety of nonheadache-related pain conditions. Its use in CH has been evaluated by Hering-Hanit and Gadoth [12] in an open-label series of 16 patients with ECH. Patients were treated with a 15–30 mg daily dose of baclofen for a 3-week period. Twelve of the 16 patients reported that their attacks ceased within a week of starting treatment, and one more reported pain freedom by week two. No side effects were reported and the drug seemed to retain its efficacy on repeated use. Tizanadine, an alpha-2 adrenergic agonist, has similar antinociception actions to baclofen in other conditions but is better tolerated. There is no published evidence of its usefulness in CH but our local experience is of a similar efficacy to baclofen. Again, randomized controlled trials are indicated on both drugs.

Valproic acid (valproate) is a drug used in the treatment of migraine and it has also been evaluated in three reports on CH treatment. Hering and Kuritzky [13] conducted an open-label study on 15 patients using 600 mg–2 g daily of valproate taken until the expected end of their bout (from 2 weeks to 2 months). Eleven patients reacted favorably to treatment; nine were rendered pain free [13]. Although patients reported typical bouts lasting many weeks, there is a possibility that spontaneous remission accounted for the positive response seen in the group. Therefore, El Amrani et al. [14] did a multicenter, double-blind, placebo-controlled study of 96 patients given 1–2 g/day of sodium valproate for a 2-week treatment period. No difference was observed between the placebo and treatment groups. The response rate in the placebo group was high (62 %), and authors state that this was likely to be due to spontaneous remission and influenced their ability to draw conclusions about the efficacy of the valproate [14]. Until further large-scale, randomized studies are conducted, the use of valproate in CH is questionable.

Candesartan was investigated by Tronvik et al. [15] in a placebo-controlled, multicenter study. Forty patients with ECH were enrolled but the trial was negative [15]. Given that post hoc analysis showed a difference in the number of attacks in the treatment group, larger studies may prove useful.

Levetiracetam is an antiepileptic drug used for partial and generalized seizures. It has been trialed in neuropathic pain and migraine, with low-quality evidence suggesting its usefulness in both conditions. Recently, Palermo et al. [16] reported two cases of ECH with attacks lasting several months responding to 1 g daily of levetiracetam given for a 4-month period. Both patients were pain free within a week and remained so 6–8 months following discontinuation of the drug. The same group also reported unpublished data of three patients with CCH responding to 2 g daily of levetiracetam. Our local experience is positive, with 3/5 patients treated over the last 6 months responding well, and two failing to respond owing to an inability to tolerate the drug’s side effects of apparent weight gain and worsening of headaches (unpublished data). Overall, the drug has a very good side effect profile and a low potential for interactions. Although the mechanism of action is unclear, initial open-label data are promising; however, further randomized controlled data are clearly needed.

#### Injectable Treatments

Botulinum toxin has been reported to relieve pain in a number of conditions, including headache, and has a licence for use in chronic migraine in the USA and Europe. Only one open-label study has looked at the effect of botulinum toxin in CH. In this study, Sostak et al. [17] treated 12 patients with CH (9 with CCH) with a total of 50 units injected into the temporalis, frontalis, splenium capitis, and trapezius ipsilateral to the headache. A reduction in attack frequency was seen by 3/12 patients, all with CCH, with a response lasting 2–3 months postinjection. Case reports exist in abstract form for 12 patients. These patients were injected with 24–50 units into the temporalis and frontalis muscles [18–20]. Eight patients are reported to have had an improvement, with four patients with ECH having abrupt cessation of their attacks. Although botulinum toxin is an attractive treatment option owing to its potential long action, lack of side effects, and drug interactions, further controlled trials similar to those done in chronic migraine are essential.

#### Nerve Blocks

Transitional treatments are required when trying to gain rapid control of attacks either during a short bout or until a preventative agent takes effect. The standard option is oral corticosteroids but these should be prescribed with caution (in our unit a maximum of twice a year) owing to the risk of side effects with prolonged or frequent use. The alternative treatment option is peripheral nerve blockade using local anesthetics, often in combination with corticosteroids.

Occipital nerve blocks are the most widely used and evaluated nerve block in the treatment of headache. The greater (GONB) and lesser occipital nerve blocks have been targeted owing to the physiological connections between the upper cervical nerves and the trigeminal nerves in the trigeminocervical complex. The majority of publications are of case reports or observational studies. The most recent of these, by Lambru et al. [21], looked at the efficacy of GONB in CCH and found that after the first injection of methylprednisolone and lidocaine, 57 % of patients reported a beneficial response lasting, on average, 21 days. These results were reproduced on repeated injections. Transient worsening of headaches was seen in 6 % of patients.

Two groups have published prospective placebo controlled data. Leroux et al. [22•] randomly allocated 43 patients (15 CCH, 28 ECH) to treatment with three suboccipital injections of steroid given over 72 h or placebo. A significant difference was found between treatment groups in the number of attacks post-treatment, in the time taken to remission, and in patient satisfaction with treatment [22•]. A reduction in the dose of additional verapamil and rescue medication taken was also seen. Ambrosini et al. [23•] conducted a blinded placebo-controlled trial on 23 patients. A single injection of suboccipital steroid led to complete attack suppression in 80 % of patients, with remission lasting at least 4 weeks.

Safety data on GONB are, on the whole, favorable; however, it is as yet unclear as to whether the blocks are better given as a series or a single injection. In the routine clinic setting it is often difficult to organize repeated injections, and larger studies would be needed to change the current practice of single injections.

Other sites used for nerve blocks include supraorbital, auriculotemporal and supratrochler blocks, although no controlled evidence exists for their use. Sphenopalatine ganglion (SPG) blockades have been used to terminate CH since the early 1900s but are clinically difficult to perform. Devoghel [24] published a series of 120 blocks and reported a response rate of 85 %. Pipolo et al. [25] reported a series of 15 patients undergoing endoscopic SPG block for CH in which 54 % experienced complete remission for between 1 and 28 months. Although not widely used, this and the increasingly positive data emerging from SPG stimulation, may mean that SPG blocks should be considered as part of the treatment arsenal for refractive patients.

#### Resectional and Ablative Surgery

The disabling nature of CH has led many surgeons to target the trigeminal nerve in a bid for pain control. Trigeminal nerve root section was the traditional option for refractory CCH prior to the advent of neuromodulation. Jarrar et al. [26] reported that 76 % of their 17 patients with CCH experienced long-term full or near complete pain relief. However, adverse effects were dramatic, with one death, cerebrospinal fluid leaks, and a case of meningitis. Another widely reported adverse outcome of this type of surgery is corneal anesthesia; in the series of Jarrar et al., two patients needed surgery for this complication in order to prevent blindness. Other groups have reported negative outcomes with CH continuing after trigeminal nerve root section [27].

Kano et al. [28] reviewed stereotactic Gamma knife radiosurgery of the trigeminal and SPG for CCH in 2011 and concluded that Gamma knife for CCH was a minimally invasive and potentially attractive alternative to nerve root section, with 60 % of patients reporting long-lasting pain relief. However, they found that CCH patients were far more likely to suffer facial sensory disturbances postoperatively than those with trigeminal neuralgia (50 % compared with 10 %) [28]. A similar review by McClelland et al. [29] was less positive, finding that any initial benefit regressed with time, leaving none of their nine patients reporting >50 % improvement by 2 weeks–2 years post-treatment, with 50 % of patients suffering some trigeminal nerve dysfunction as a consequence of surgery. However promising the immediate results of trigeminal nerve surgery may appear, the adverse effects and long-term outcomes are consistently poor, and with neuromodulation producing favorable results in terms of safety and follow-up, this sort of surgery should very much be at the bottom of any treatment list, if conducted at all.

#### Neurostimulation

Occipital nerve stimulation (ONS) is a nondestructive surgical option for refractory CCH with an increasing evidence base of open-label studies. Burns et al. [30•] first examined the role of ONS in CCH in a series of 14 patients. Ten reported benefit, three a benefit of >90 %, and three of >40 %. Magis et al. [31] followed up 14 patients over an average of 37 months and reported 11 to be receiving an at least 90 % reduction in attacks. From these and other series available, ONS appears to be safe, with the most frequent adverse effects reported being dependent on surgical experience and battery depletion, which is overcome by the introduction of rechargeable batteries. A number of series report a side-shift in attacks if unilateral stimulation is employed, and so bilateral lead placement is now standard. A review of the current literature on ONS is given by Magis and Schoenen [32••].

The SPG has also been a target for neuromodulation owing to its links with the trigeminovascular system. Ansarinia et al. [33] investigated SPG stimulation in six patients and found that complete resolution of pain was seen within 3 mins in 11/18 attacks treated. A larger, multicenter, sham-controlled study was published in 2013 on the use of on-demand SPG stimulation. A novel device was implanted in 28 patients who randomly received full, sham, or subperception stimulation. There was a significant difference in the number of resolving attacks in the treated group. Sixty eight percent of treated patients had clinically significant resolution of attacks. Adverse events were mild, with 81 % reporting transient facial sensory disturbance and only five cases requiring surgical revision for lead migration or explanation [34••]. Unexpectedly, many patients demonstrated a reduction in attack frequency following regular use of SPG stimulation despite the study being designed to treat acute attacks. Further work is ongoing into the prophylactic efficacy of SPG stimulation.

Deep brain stimulation (DBS) in CCH stems from imaging findings showing the posterior hypothalamus becoming overactive in attacks [35]. Leone et al. [36] reported the first case in 2001, and there are now >60 published cases. A recent review of DBS was included in the review by Magis and Schoenen [32••]. The overall clinical success rate is around 66 % (mean follow-up 2 years) with a mean delay to clinical result of 42 days. The only placebo-controlled trial of DBS randomized 11 patients to active or sham stimulation for 2 months. No difference was found between groups [37], but, given the findings of other series of time delays to response of 1–86 days, this is thought likely to be due to the short treatment phase. The most commonly reported adverse effects are transient dizziness and visual disturbance associated with amplitude changes.

However, DBS is not without serious risk, with one patient dying from intracerebral hemorrhage postoperatively [38]. With collated data on DBS for CCH the risk for serious hemorrhage is 3 %, which is within the range reported for DBS for movement disorders [32••]. Owing to the risks of surgery and lack of controlled evidence, the European Headache Federation has reviewed the use of neuromodulation in CCH. It has recommended that procedures should only be considered when all other medical treatments have failed, should be carried out by tertiary headache centers, and should start with the least invasive methods before considering DBS [39].

## PH

PH is characterized by attacks of severe, strictly unilateral pain occurring in the orbital or temporal regions that last for 2–30 minutes and occur several or many times a day accompanied by autonomic symptoms. The condition responds completely to indometacin (Table 1) [1].

Acute medications are often of little use in this condition as the attacks are too short lasting for any to take effect.

### Preventative Medication

#### Oral Drugs

By definition, PH must show an absolute response to indometacin and the diagnosis reconsidered if this response is not seen [1]. Likewise, any patient requiring escalating doses or suddenly becoming unresponsive to indometacin should be reviewed for other pathology. The main reason to use alternative treatments in this condition is the poor tolerability of long-term indometacin, with 30 % of patients developing gastrointestinal (GI) side effects (Table 3) [40].

**Table 3:** Summary of evidence for oral and injectable alternative treatments for paroxysmal hemicrania (PH) and hemicrania continua (HC\0

	*n*	Outcome	Dosing details
PH			
COX-2 inhibitors			
Celecoxib			
Mathew et al. [41]	1	Positive with 3-month follow-up	400 mg daily
Rofecoxib			
Lisotto et al. [42]	2	Positive 2/2 with 8–14-month follow-up	25–50 mg daily
Topiramate			
Cohen et al. [43], Camarda et al. [44]	2	Positive with 6-month–2-year follow-up	25–200 mg daily
Verapamil			
Evers et al. [45]	10	Positive 4/10	Maximum 320 mg daily
Flunarazine			
Coria et al. [46]	5	Positive 5/5 with 6-month follow-up	
Nerve blocks			
GON			
Rossi et al. [47]	1	Positive to 5 injections over 20 months	Methylprednisolone and lidocaine
GON, LON, or SON			
Antonaci et al. [48]	6	Negative in all	Lidocaine
HC			
COX-2 inhibitors			
Rofecoxib			
Peres et al. [52], Muller et al. [51]	10	Positive 4/9	25–50 mg daily
Celcoxib			
Peres et al. [52]	5	Positive 4/5	800 mg daily
Melatonin			
Rozen [53], Spears [54], Hollingworth et al. [55]	5 (1 patient not trial indometacin)	Positive 5/5 with 3–5-month follow-up	9–15 mg daily
Topiramate			
Camarda et al. [44], Matharu et al. [56], Brighna et al. [57], Prakash et al. [58]	6	Positive with follow-up of 6 weeks–1 year	100–200 mg daily
Verapamil			
Rozen [59], Rajabally and Jacob [60]	2	Positive with follow-up of 8–9 months	120–480 mg daily
Gabapentin			
Prakesh et al. [58], Moura et al. [61], Spears [62]	12	Positive 10/12 with 2–13-month follow-up	600–3600 mg daily
Nerve blocks			
SON, trochlear, GON, LON			
Guerrero et al. [63]	9	Positive 9/9 (5/9 pain free)	Bupivacaine, mepivacane, or triamcinolone
GON, LON, or SON			
Antonaci et al. [48]	7 at each site	Positive 4/7 SON, negative GON and LON	Lidocaine
Botulinum toxin			
Khali et al. [64], Garza and Cutrer [65]	2	Positive over 3–4 injection cycles	100–155 units

*COX-2* cyclooxygenase 2, *GON* greater occipital nerve, *LON* lesser occipital nerve, *SON* supraorbital nerve

Cyclooxygenase (COX)-2 inhibitors are reported to have a lower rate of GI side effects but should still be used with caution in those with peptic ulcer disease. Case series from two groups have shown some efficacy using celecoxib and rofecoxib [41, 42]. Case reports also support the use of topiramate, with two patients reporting continued remission after 2 years [43, 44]. Calcium channel blockers have also been trialed in open-label series. Verapamil was found by Evers and Husstedt [45] to have a mild-to-moderate effect, and flunarazine induced complete remission in the five patients treated by Coria et al. [46], with no reported side effects.

#### Nerve Blocks

The use of GON blocks in PH is supported by a single case report by Rossi et al. [47]. Antonaci et al. [48] conducted blocks of the greater occipital, lesser occipital, and supraorbital nerves in six patients with chronic PH with a negative effect in all. Despite the limited evidence, GON blocks are widely employed in headache clinics for PH with some success. Obviously, larger studies are needed to support this practice.

#### Neurostimulation

Once again, there is very little published evidence on the use of neurostimulation for PH. No reports exist for the use of ONS in PH; however, our local experience is that it may be useful in refractory patients (unpublished data).

Walcott et al. [49] have published a single case of DBS in PH. The patient had an immediate and complete response to DBS, with the pain returning within hours after stopping DBS. With a follow-up period of 2 years, the device had retained its efficacy. On the basis of such limited evidence, neurostimulation should only be considered in patients who have failed all possible medical treatments mentioned above.

## HC

It is only recently that HC has been classified as a TAC, having previously been considered a separate primary headache disorder. The disorder is characterized by persistent, strictly unilateral head pain associated with autonomic features, and that is completely responsive to indometacin (Table 1) [1].

Acute medications, including oxygen and triptans, have generally been reported to be of little use in this condition [50].

### Preventative Treatments

#### Oral Treatments

By definition, HC must show a complete response to indometacin and the diagnosis reconsidered if this is not seen. The poor tolerability of indometacin is the main reason alternatives are sought (Table 3).

Once again, the evidence is limited to case reports, but oral drugs that may prove useful include COX-2 inhibitors (Rofecoxib and Celecoxib) [51, 52], melatonin [53–55], topiramate [44, 56–58], verapamil [59, 60], and gabapentin [58, 61, 62].

#### Injectable Treatments

The use of nerve blocks in HC is supported by two case series using multiple cranial nerve blocks. Guerrero et al. [63] used combinations of supraorbital, trochlear, and greater and lesser occipital nerve blocks with good effect, and Antonaci et al. [48] suggested that the positive response of HC to supraorbital nerve blocks could be used to differentiate it from PH. Antonaci et al. [48] found that GONBs were ineffective in their patient group; however, in our experience, patients often respond well and our practice is to offer this treatment.

Limited case reports exist for the efficacy of botulinum toxin in HC [64, 65]. However, our local experience of good results in 5/7 patients suggests that it can be useful and should be considered in resistant cases (unpublished data).

#### Neurostimulation

There are three case series and reports of neuromodulation in HC. Burns et al. [66] and Schwedt et al. [67] implanted a BION device in a total of seven patients, with five reporting >50 % improvement. Schwedt et al. [68] also included two HC patients in their series of 15 ONS implantations and although individual results for this headache type is not given in the paper, the group quote a mean reduction of headache frequency of one-third across the series.

## Short-Lasting Unilateral Neuralgiform Headache Attacks

These conditions consist of attacks of moderate or severe, strictly unilateral head pain lasting seconds to minutes, which occur at least once a day and are usually associated with prominent lacrimation and redness of the ipsilateral eye [1]. Two subtypes are recognized in the International Classification for Headache Disorders-3 beta: SUNCT and SUNA. The differentiation is made on the associated autonomic features; patients with SUNCT exhibit both conjunctival injection and tearing, whereas patients with SUNA will have only one or neither of them (Table 1). Attacks are so brief that acute medication is of little use to patients.

### Preventative Medication

#### Oral Treatments

Current guidelines for the preventative treatment of short-lasting unilateral neuralgiform headache attacks advise first-line treatment with lamotrigine [2, 69]. Other oral therapies, including topiramate, gabapentin, oxcarbazepine, carbamazepine, and mexiletine all have case reports to support their use as alternative agents (Table 4).

**Table 4:** Summary of evidence for oral and injectable alternative treatments for short-lasting unilateral neuralgiform headache attacks

	*n*	Outcome	Dosing details
Topiramate			
Kuhn et al. [70], Matharu et al. [71], Rossi et al. [72]	4	Positive 4/4 with follow-up of 5–9 months	50–200 mg daily
Gabapentin			
Etemadifar et al. [73]	8	Positive 8/8 (5 pain free)	600–900 mg daily
Carbamazepine			
Matharu et al. [74]^a^	33	Complete or partial response 11/33 (alone or combination)	600–3000 mg daily
Oxcarbazepine			
Marziniak et al. [75], Dora [76]	2	Positive 2/2 with follow up 1–36 months	600 mg daily
Intravenous lidocaine			
Matharu et al. [77]	4	Positive 4/4	1–3 mg/min
Botulinum toxin			
Zabalza [79]	1	Positive after 3 years of 3-monthly injections	Injections around orbit
Nerve blocks			
GON			
Porta-Essam et al. [80]	1	Positive	Bupivacaine
Pareja et al. [81]	3	Negative	Lidocaine
SON			
Pareja et al. [81]	7	Negative	Lidocaine

*GON* greater occipital nerve, *SON* supraorbital nerve

^a^Review article

Kuhn et al. [70], Matharu et al. [71], and Rossi et al. [72] have reported a total of four patients achieving freedom from pain on low doses of topiramate. Our experience is that higher doses are often required to gain control of the condition.

Gabapentin has been reported in one open-label study and three case reports to be efficacious in short-lasting unilateral neuralgiform headache attacks. The open-label study conducted by Etemadifar et al. [73] consisted of eight patients all who responded well, with five becoming pain free on 600 mg daily.

Carbamazepine is first-line treatment for trigeminal neuralgia, and given the clinical similarities and high misdiagnosis rates between conditions a number of patients have been trialed successfully on the drug in the clinical setting. Matharu et al. [74] reviewed 33 patients given carbamazepine. Twenty-two had no response and only three patients reported a complete response to carbamazepine either alone or in combination with other drugs. Oxcarbazepine, a structural derivative of carbamazepine, is a drug we frequently use locally for short-lasting unilateral neuralgiform headache attacks as it appears to be well tolerated and as efficacious as carbamazepine. However, only two case reports exist in the literature to support its use [75, 76].

#### Injectable Treatments and Nerve Blocks

Intravenous lidocaine is a useful agent in the treatment of severe attacks or short-lasting unilateral neuralgiform headache attack status. Matharu et al. [77] reported a series of four intractable patients rendered pain free by the infusion. The use of lidocaine and mexiletine, both antiarrthmyic drugs acting on sodium channels, are reviewed in a paper by Marmura [78].

Limited reports exist on the use of botulinum toxin and GONB for short-lasting unilateral neuralgiform headache attacks [79–81]. Our local experience is that GONB can be useful in a small subset of patients.

#### Resectional and Ablative Surgery

A number of procedures have been conducted over the years, often under the misdiagnosis of trigeminal neuralgia, including microvascular decompression, glycerol rhizotomy, trigeminal nerve radiofrequency ablation, balloon compression of the Gasserion ganglion, and Gamma knife therapy. Outcomes are mixed but, in general, the adverse effects outweigh any benefit. The one procedure that has appeared in recent reviews to be efficacious, even with long-term follow-up, is microvascular decompression (MVD) of the trigeminal nerve. An Australian group found 19 patients described in the literature undergoing MVD. After a 14-month follow-up, 63 % had complete resolution of pain and the rest had little or no change. Adverse effects included wound infection, vertigo, and jaw pain, and more serious effects of persistent hearing loss and ataxia [82••]. Larger patient numbers and follow-up periods are needed, but MVD appears a sensible option in refractory patients with an aberrant vessel on imaging before attempting neuromodulation, which is more expensive and invasive.

#### Neurostimulation

A case series of nine patients undergoing ONS for SUNCT/SUNA was reported by Lambru et al. [83], with eight reporting a positive response and four freedom from pain. Supraorbital and supratrochlear stimulation has also been successful in one patient [84].

The outcomes of DBS for SUNCT appear promising, with three patients gaining substantial relief within a few months of surgery [85–88]. Our own data consist of 5/6 patients treated responding well, with two achieving freedom from pain [89].

## Conclusion

CH, PH, HC, and short-lasting unilateral neuralgiform headache attacks are well-defined stereotyped syndromes that can be differentiated by their clinical features and treatment responses. Whilst each syndrome has standard treatment options, there will be a small but disabled group of patients who will be unresponsive to these and require alternative agents. The evidence base for any of these alternative treatments is poor, but a wide variety of oral, injectable, and surgical options does exist. Table 5 summarizes the alternative options used in our clinic. Surgical techniques should be reserved for patients proving intractable to all available medical treatments and, with the exception of SUNCT/SUNA treated with MVD, neurostimulation techniques will be the choice in the future. One treatment that should potentially be made more widely available to TAC patients is GONB. On the basis of highly positive CH data, the favorable safety profile, and the ease of the procedure, a strong argument can be made for using nerve blocks earlier in the treatment regime or as a bridging therapy in many more patients.

**Table 5:** Suggested alternative treatments for trigeminal autonomic cephalalgias

	Cluster headache	Paroxysmal hemicrania	Hemicrania continua	Short-lasting unilateral neuralgiform headache attacks
Oral treatments	Melatonin: 12–16 mg daily Gabapentin: 3600 mg daily Baclofen: 90 mg daily Levitracetam: 2–3 g daily	COX-2 inhibitors (celecoxib: 400 mg daily; etoricoxib 900 mg daily) Topiramate: 400 mg daily Verapamil: 960 mg daily Flunarazine: 10 mg daily	Celecoxib: 400 mg daily Topiramate: 400 mg daily Gabapentin: 3600 mg daily Melatonin: 15–16 mg daily Verapamil: 960 mg daily	Oxcarbazepine: 1500 mg daily Carbamazepine: 1200 mg daily Topiramate: 400 mg daily Gabapentin: 3600 mg daily Mexiletine: 1200 mg daily
Nerve blocks	GONB^a^ MCNB^b^	GONB^a^ MCNB^b^	GONB^a^ MCNB^b^	GONB^a^ MCNB^b^
Surgery	ONS DBS SPG	ONS DBS	ONS DBS	MVD ONS DBS

*COX* cyclooxygenase, *GONB* greater occipital nerve block, *MCNB* multiple cranial nerve block, *ONS* occipital nerve stimulation, *DBS* deep brain stimulation, *SPG* sphenopalatine ganglion stimulation, *MVD* microvascular decompression

^a^GONB: 2 ml 2 % lidocaine and 80 mg methylprednisolone

^b^MCNB: greater occipital nerve 80 mg methylprednisolone, 1 ml bupivocaine 0.5 %, and 1 ml lidocaine 2 %; lesser occipital nerve supraorbital, supratrochlear, and auriculotemporal 50:50 mix lidocaine 2 % and bupivocaine 0.5 %

Currently, the most robust evidence exists for the treatment of CH with the use of GONB, SPG stimulation, and melatonin. DBS appears promising but this is on the basis of increasing numbers of case reports only. It is clear that large randomized trials are needed on all TAC treatments to allow guidelines to be based on efficacy and safety.
